# Transcription Factors *Sp8* and *Sp9* Regulate Medial Ganglionic Eminence-Derived Cortical Interneuron Migration

**DOI:** 10.3389/fnmol.2019.00075

**Published:** 2019-04-02

**Authors:** Guangxu Tao, Zhenmeiyu Li, Yan Wen, Xiaolei Song, Song Wei, Heng Du, Zhengang Yang, Zhejun Xu, Yan You

**Affiliations:** State Key Laboratory of Medical Neurobiology, MOE Frontier Research Center for Brain Science, Department of Neurology, Institutes of Brain Science, Zhongshan Hospital, Fudan University, Shanghai, China

**Keywords:** *Sp8*, *Sp9*, medial ganglionic eminence, cortical interneuron, tangential migration, parvalbumin, somatostatin

## Abstract

Cortical interneurons are derived from the subpallium and reach the developing cortex through long tangential migration. Mature cortical interneurons are characterized by remarkable morphological, molecular, and functional diversity. The calcium-binding protein parvalbumin (PV) and neuropeptide somatostatin (SST) identify most medial ganglionic eminence (MGE)-derived cortical interneurons. Previously, we demonstrated that *Sp9* plays a curial transcriptional role in regulating MGE-derived cortical interneuron development. Here, we show that SP8 protein is weekly expressed in the MGE mantle zone of wild type mice but upregulated in *Sp9* null mutants. PV^+^ cortical interneurons were severely lost in *Sp8/Sp9* double conditional knockouts due to defects in tangential migration compared with *Sp9* single mutants, suggesting that *Sp8/9* coordinately regulate PV^+^ cortical interneuron development. We provide evidence that *Sp8/Sp9* activity is required for normal MGE-derived cortical interneuron migration, at least in part, through regulating the expression of *EphA3*, *Ppp2r2c*, and *Rasgef1b*.

## Introduction

The cerebral cortex plays an irreplaceable role in many advanced functions, such as learning, movement, emotion memory and decision-making. The normal execution of these functions depends on the subtle ratio of excitatory projection neurons to inhibitory interneurons in the cerebral cortex and the appropriate functional circuits formed between them (Rubenstein and Merzenich, 2003; Del Pino et al., 2018; Lim et al., 2018). Glutamatergic cortical excitatory projection neurons are generated by pallial (cortical) neuroepithelial and radial glial cells (Kriegstein and Alvarez-Buylla, 2009). On the other hand, GABAergic cortical inhibitory interneurons, both in primates and rodents, are derived from the subpallium and reach their final position in the cortex through tangential migration (Wonders and Anderson, 2006; Hansen et al., 2013; Ma et al., 2013). During development, the subpallium is composed of four major proliferative zones: the medial (MGE), lateral (LGE) and caudal (CGE) ganglionic eminences and the preoptic area (POA); the MGE generates 60% of all cortical interneurons and includes parvalbumin (PV^+^) and somatostatin (SST^+^) subtypes (Gelman and Marín, 2010; Hu et al., 2017; Wamsley and Fishell, 2017).

The *Sp9* zinc finger transcription factor is widely expressed in the ganglionic eminences (Long et al., 2009; Zhang et al., 2016). *Sp8* and *Sp9* are two closely related button head-like transcription factors and have many redundant functions in regulating GABAergic neuronal development (Li et al., 2018; Xu et al., 2018). Previously, we showed that *Sp9* has a curial transcriptional role in regulating MGE-derived cortical interneuron development (Liu et al., 2018). While *Sp8* is highly expressed in the dorsal LGE and the CGE (Ma et al., 2012), it is also weekly expressed in the MGE (Vogt et al., 2014). However, the function of *Sp8* in the MGE remains largely unknown.

In the present study, we found that SP8 protein expression was upregulated in the MGE mantle zone of *Sp9* null mutants. There were more MGE-derived cortical interneurons that failed to migrate into the cortex in *Sp8/Sp9* double conditional knockouts than in *Sp9* single conditional knockouts. Furthermore, fewer *Erbb4*^+^ cells (immature PV^+^ cortical interneurons) migrated in the cortical marginal zone (MZ), whereas more migrated in the cortical subventricular zone (SVZ). In the postnatal cortex, the most prominent phenotype was that approximately 80% of PV^+^ interneurons were lost in *Sp8/Sp9* double conditional knockouts. We provide evidence that *Sp8* and *Sp9* mediate these effects by regulating the expression of several genes that regulate cortical interneuron migration and development, such as *EphA3*, *Ppp2r2c* and, *Rasgef1b*.

## Materials and Methods

### Mice

All animal experiments described in this study were approved in accordance with institutional guidelines, and the institutional review board (Ethics Committee) of Shanghai Medical College of Fudan University approved the study design. The strains used in this study have been previously reported: *Nkx2-1-Cre* (Xu et al., 2008)*, Rosa26-YFP (R26R-YFP*; Srinivas et al., 2001), *Sp9*^*LacZ/+*^ (Zhang et al., 2016), *Sp9*^F/+^ (Zhang et al., 2016), *Sp8*^F/+^, (Bell et al., 2003) and *Lhx6-Cre* (Fogarty et al., 2007). All lines were maintained in a mixed genetic background of C57BL/6J, 129 and CD1. Pregnancy of mated mice was determined by vaginal plug detection, which was defined as embryonic day 0.5 (E0.5), and the day of birth was defined as postnatal day 0 (P0). The range of embryos used had a ±0.5-day deviation.

### Tissue Preparation

The pregnant mice were killed by cervical dislocation on designated dates. Each embryo was separated from the placenta, and the brain was dissected out and then fixed in 4% diethylpyrocarbonate and paraformaldehyde (DEPC-PFA) for several hours or overnight. Postnatal mice were deeply anesthetized and perfused adequately with cold 0.01 M PBS and 4% DEPC-PFA, and brains were then removed and postfixed overnight. Cryosectioning (Leica, CM 1950) was performed on brains embedded in optimal cutting temperature (OCT). (Sakura); brains were immersed in OCT for 5 min at 4°C and subsequently transferred to a plastic mold filled with OCT and frozen in dry ice-chilled ethanol.

### Immunohistochemistry

Immunohistochemistry was performed on 12 μm (embryonic brains) or 30 μm (postnatal brains) coronal sections (Liu et al., 2018). Briefly, sections were blocked for 30 min in TBS with 0.1% Triton X-100 and 5% donkey serum. For double staining, sections were incubated simultaneously with primary antibodies from different species, and secondary antibodies were used sequentially. Primary antibodies were incubated for 24 h at 4°C. We used rabbit anti-calretinin (CR; 1:3,000, AB5054, Millipore, Burlington, MA, USA), chicken anti-GFP (1:2,000, GFP-1020, Aves Labs), rabbit anti-PV (1:2,000, PV25, Swant), rabbit anti-NKX2-1 (1:500, sc-13040, Santa Cruz Biotechnology, Dallas, TX, USA), rabbit anti-NPY (1:500, 22940, Incstar), goat anti-SST (1:500, sc-7819, Santa Cruz Biotechnology, Dallas, TX, USA) and goat anti-SP8 (1:2,000, sc-104661, Santa Cruz Biotechnology, Dallas, TX, USA). Secondary antibodies (1:400, Jackson Immuno Research) were incubated for 2 h at room temperature (RT), rinsed three times in TBS, and then incubated with DAPI (4′,6-diamidino-2-phenylindole, 1:5,000) for 3 min.

### *In situ* Hybridization

RNA *in situ* hybridization experiments were performed using digoxigenin riboprobes on 20 μm cryostat sections (Liu et al., 2018; Xu et al., 2018). The previously described full-length cDNA probe of SST was used (McKinsey et al., 2013). Templates for making other riboprobes were amplified by PCR using the following primers:

**Table:** 

(1) *Erbb4*	Fwd: GCACCGATATTTGCCCCAA
	Rev: CAGTCATGACTAGTGGGACCGTTAC
(2) *EphA3*	Fwd: TGTATGGAGTTACGGGATTGTTC
	Rev: GGCTCTACACTAGTTCTTCCACTTC
(3) *Ppp2r2*c	Fwd: CGGACGACCTACGCATCAACCT
	Rev: GCCCTGCCTCACGATTAACCCTA
(4) *Rasgef1b*	Fwd: GTGGCTACAACCGAAACCTCTA
	Rev: AGACCGGGCTCATATTCATACC
(5) *Npas1*	Fwd: CGTGCGTCTTAGCGTCACCTACC
	Rev: GCCTCCACTTTGATGCGTTTGC

### Microscopic Imaging

Immunofluorescence staining images were taken using an Olympus BX51 metallographic microscope, an Olympus VS120 digital slice scanning system or an Olympus FV1000 laser scanning confocal microscope. Brightfield images (*in situ* hybridization) were taken using an Olympus BX51 metallographic microscope. Images were manipulated with Photoshop CS5 software (Adobe Systems, San Jose, CA, USA). The contrast and brightness of these images were adjusted for better visualization.

### Cell Counting and Data Collection

DAPI staining was used to demarcate MZ, cortical plate (CP), subplate (SP), intermediate zone (IZ), SVZ, and VZ. The numbers of GFP^+^ cells in the E15.5 cortex were counted in 250-μm-wide bins that spanned from the MZ to the VZ. The percentage of GFP^+^ cells in each layer was calculated. And the numbers of *Sst*^+^, *Erbb4*^+^, *Npas1*^+^ cells in the E15.5 cortex were counted in 350-μm-wide bins that spanned from the MZ to the VZ. We counted cells from three sections of each mouse and analyzed three mice of each genotype [*Nkx2-1-Cre; Rosa-YFP* (controls), *Nkx2-1-Cre; Sp9^F/F^; Rosa-YFP* (*Nkx2-1-Cre; Sp9-CKOs*); *Nkx2-1-Cre; Sp8^F/F^; Sp9^F/F^; Rosa-YFP* (*Nkx2-1-Cre; Sp8/9-DCKOs*)]. For P30 mice, the numbers of GFP^+^, GFP^+^/PV^+^, GFP^+^/SST^+^, GFP^+^/NPY^+^, and GFP^+^/CR^+^ cells in the somatosensory cortex were analyzed in 1,000-μm-wide bins (*n* = 3 mice for each genotype, three sections per mouse).

IBM SPSS Statistic 22 and GraphPad Prism 6 was used for statistical analysis, and all data are expressed as the means ± standard error. For comparisons of means between groups, *t*-test, One-way analysis of variance (ANOVA), the least significant difference (LSD) test, the Bonferroni correction, the Student–Newman–Keuls (SNK) test, Tamhane’s T2 test, and Dunnett’s T3 test were used; *P* < 0.05 was considered significant (*P < 0.05; **P < 0.01; ***P < 0.001).

## Results

### SP8 Expression Is Upregulated in the MGE Mantle Zone of *Sp9* Null Mutants

**Figure 1:**
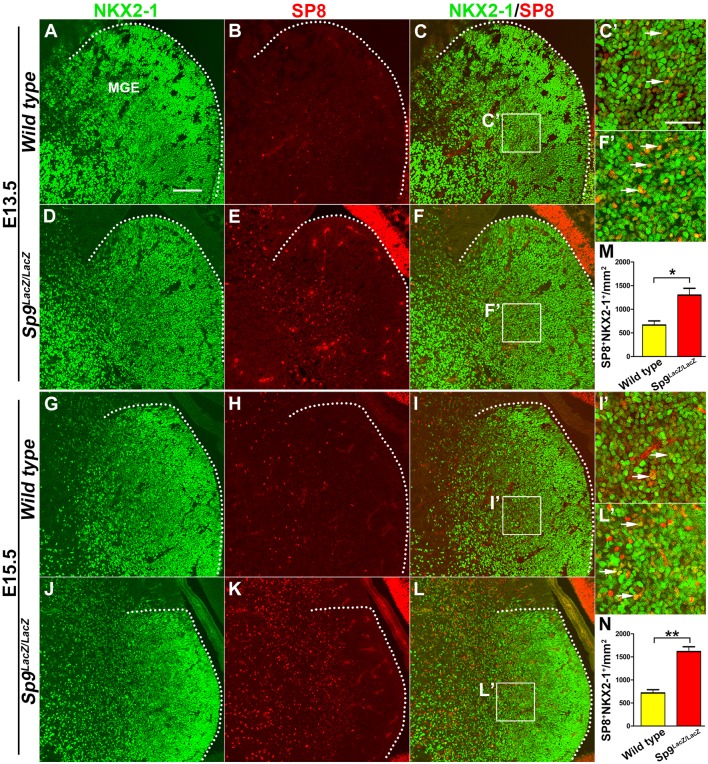
SP8 expression is upregulated in medial ganglionic eminence (MGE) of *Sp9^LacZ/LacZ^* null mutants. **(A–L)** NKX2-1/SP8 double-immunostained coronal sections at E13.5 and E15.5. **(C′,F′,I′,L′)** Higher magnification images of the boxed areas in **(C,F,I,L)**. Upregulation of SP8 protein expression can be noted in the mutant MGE mantle zone; most of these SP8^+^ cells exhibit NKX2-1 coexpression. **(M,N)** Quantified data showed that the density of SP8^+^/NKX2-1^+^ cells was increased in *Sp9^LacZ/LacZ^* mutants compared with controls in E13.5 and E15.5. Scale bars: 100 μm in **(A)** for **(A–L)**; 50 μm in **(C’)** for **(C′,F′,I′,L′)**.

SP9 is strongly expressed in the SVZ of the MGE, but very few SP9^+^ cells are observed in the MGE VZ (Liu et al., 2018). SP8 is also not expressed in the MGE VZ and SVZ, but we observed that a subset of cells in the MGE mantle zone weakly expressed SP8 protein at E13.5 and E15.5 (Figures 1A–C′,G–I′). This finding is consistent with a previous report, which showed that *Sp8* mRNA is weakly expressed in the MGE (Vogt et al., 2014). In the Sp9^*LacZ/LacZ*^ null mutant, however, SP8 expression was greatly upregulated in the MGE mantle zone and in a subset of MGE-derived cells (Figures 1D–F′,J–L′; Liu et al., 2018). We next quantified the number of NKX2-1^+^/SP8^+^ cells in the MGE mantle zone. The *Sp9* mutants showed >120% increase in NKX2-1^+^/SP8^+^ cells compared to wild type controls (Figures 1M,N). Given a high degree of similarity between the *Sp8* and *Sp9* genes, upregulation of SP8 expression may compensate for *Sp9* function in the MGE. To test this hypothesis, we compared MGE development in control, *Nkx2-1-Cre; Sp9-CKO* (*Sp9* single mutant) and *Nkx2-1-Cre; Sp8/9-DCKO* (*Sp8/9* double mutant) mice.

### *Nkx2-1-Cre; Sp8/9-DCKO* Mice Show Severe Defects in Tangential Migration of MGE-Derived Cortical Interneurons

**Figure 2:**
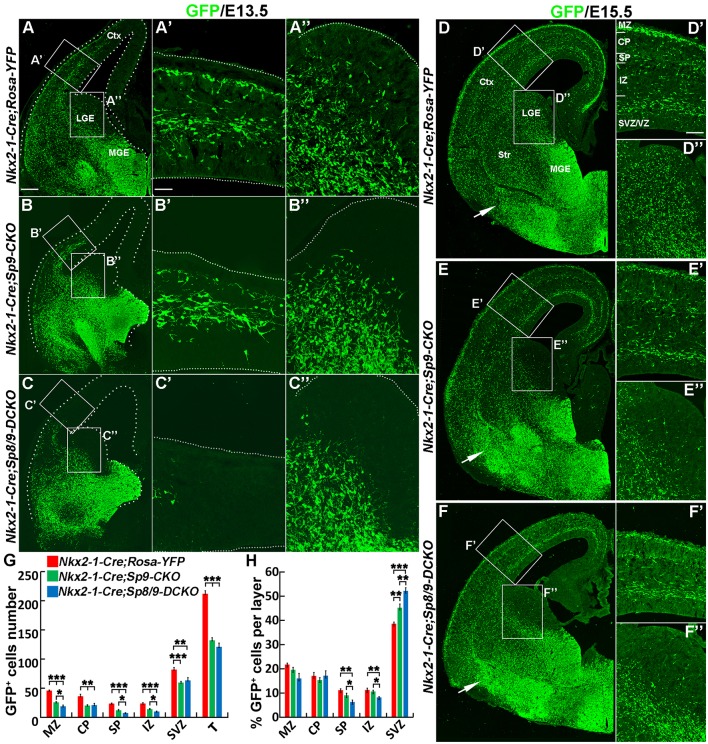
Tangential migration defects of MGE-derived cortical interneurons in *Nkx2-1-Cre; Sp8/9-DCKO* mice. **(A–C)** GFP immunostained E13.5 coronal hemisections. **(A′–C″)** Higher magnification images of the boxed areas in **(A–C)** show that MGE-derived GFP^+^ cortical interneurons migrate to the cortex much less efficiently in *Nkx2-1-Cre; Sp8/9-DCKO* (double mutant) mice than in controls and *Nkx2-1-Cre; Sp9-CKO* (single mutant) mice. Very few GFP^+^ cells can be noted in the lateral GE (LGE) subventricular zone (SVZ) of mutants **(B″, C″)**. **(D–F)** GFP immunostained E15.5 coronal hemisections. **(D′–F″)** Higher magnification images of the boxed areas in **(D–F)**. Note that more GFP^+^ cells ectopically accumulated in the ventral telencephalon in *Nkx2-1-Cre; Sp8/9-DCKO* mice than in controls and *Nkx2-1-Cre; Sp9-CKO* mice (arrows). **(G,H)** Quantification showing that mutant mice had fewer GFP^+^ cells in the cortex at E15.5. The percentage of GFP^+^ cells was reduced in the SP and the intermediate zone (IZ) and increased in the SVZ. Ctx, cortex. *P < 0.05; **P < 0.01; ***P < 0.001. Scale bars: 100 μm in **(A)** for **(A–F)**; 25 μm in **(A′)** for **(A′–C″)**; 50 μm in **(D′)** for **(D′–F′′**).

In E13.5 *Nkx2-1-Cre; Rosa-YFP* control mice, many GFP^+^ cells entered the medial region of the cortex (Figures 2A,A′). In *Nkx2-1-Cre; Sp9-CKO* single mutants, GFP^+^ cells also migrated to the cortex, although less efficiently (Figures 2B,B′; Liu et al., 2018). However, in *Nkx2-1-Cre; Sp8/9-DCKO* double mutants, GFP^+^ cells only reached the lateral cortex (Figures 2C,C′). We also found few GFP^+^ cells in the LGE SVZ of single and double mutants (Figures 2B″,C″), whereas a subpopulation of GFP^+^ cells were observed in the LGE VZ/SVZ that tangentially migrated to the cortex of control mice (Figure 2A″).

At E15.5, in control, *Nkx2-1-Cre; Sp9-CKO* and *Nkx2-1-Cre; Sp8/9-DCKO* mice, migration of cortical interneurons appears to occur primarily in two streams, in the cortical MZ and the SVZ, but *Nkx2-1-Cre; Sp9-CKO* and *Nkx2-1-Cre; Sp8/9-DCKO* mice had fewer GFP^+^ cells in the cortex (Figures 2D–F″,G). We then analyzed the distribution of MGE-derived (GFP^+^) cortical interneurons. The number of GFP^+^ cells in the MZ, CP, SP, IZ and SVZ of the cortex were greatly reduced in single and double mutants compared with controls (Figure 2G), as was the percentage of GFP^+^ cells in the SP and the IZ (Figure 2H). In contrast, the percentage of mutant GFP^+^ cells in the cortical SVZ was increased (Figure 2H). Notably, the percentage of GFP^+^ cells was notably higher in the SVZ of *Sp8/9* double mutants than in *Sp9* single mutants, and the percentage of GFP^+^ cells in the SP and the IZ was much lower (Figure 2H). Again, very few GFP^+^ cells were found in the LGE SVZ of mutants (Figures 2D″–F″). The ectopic accumulation of MGE-derived GFP^+^ cells was observed in *Nkx2-1-Cre; Sp9-CKO* mice (Liu et al., 2018), but the loss of both *Sp8* and *Sp9* function led to severe increases in GFP^+^ cells in the ventral telencephalon (Figures 2D–F). Taken together, both *Sp9* and *Sp8* promote the tangential migration of MGE-derived cortical interneurons from the MGE to the cortex and control the migration of cortical interneurons that follow distinct pathways in the cortex. In addition, loss of *Sp8* function in *Sp9* mutants further increased E15.5 cortical interneurons in the deep migration zone (SVZ).

### Molecular Defects in Tangentially Migrating *Sp8/9* Double Mutant MGE-Derived Cortical Interneurons

**Figure 3:**
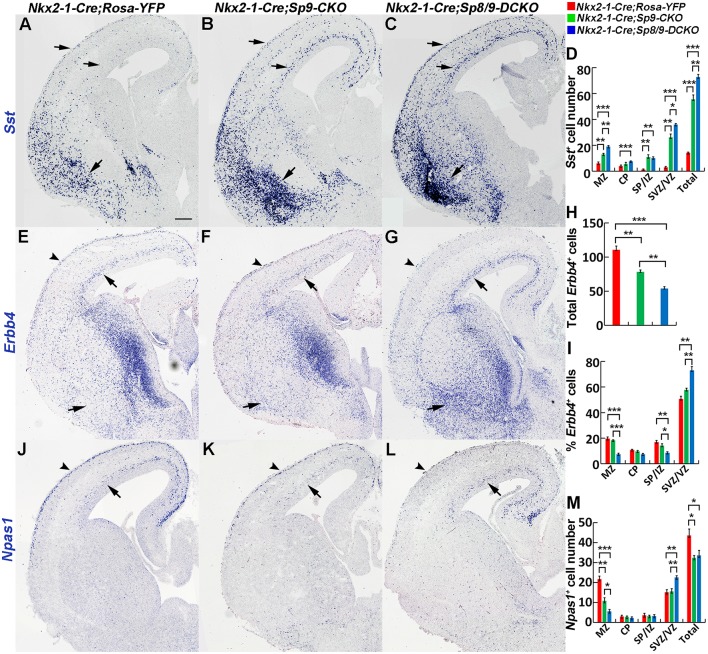
*In situ* RNA hybridization of *Sst, Erbb4* and *Npas1*. **(A–D)** *Sst* mRNA expression was increased in the E15.5 neocortex (arrows) and ventral telencephalon (arrows) of mutants. More *Sst*^+^ cells were found in the ventral telencephalon of double mutants than single mutants and controls (arrows). **(E–I)** Abnormal migration of *Erbb4*^+^ cells (PV^+^ immature cortical interneurons) in mutant mice. Double mutants appeared to have relatively more *Erbb4*^+^ cells in the cortical SVZ (arrows) and in the ventral telencephalon (arrows) and relatively fewer *Erbb4*^+^ cells in the cortical marginal zone (MZ; arrowheads). **(J–M)** Decreased *Npas1*^+^ cells in the cortical MZ (arrowheads) and increased *Npas1*^+^ cells in the cortical SVZ (arrows) were observed in double mutants compared to single mutants and controls. *P < 0.05; **P < 0.01; ***P < 0.001. Scale bar: 200 μm in **(A)** for **(A–C,E–G,J–L)**.

We next examined the molecular profile of tangentially migrating cells in these mutants at E15.5. We previously demonstrated using RNA-Seq and ChIP-Seq (chromatin coimmunoprecipitation followed by high-throughput DNA sequencing) that *Sp9* has a curial transcriptional role in regulating MGE-derived cortical interneuron development (Liu et al., 2018). Loss of *Sp9* function resulted in upregulation of *Sst* expression in a subset of MGE-derived cells; however, *Sst* expression was further upregulated in *Sp8/9* double mutants (Figures 3A–C). Indeed, although *Sp8/9* double mutants and *Sp9* single mutants had almost same numbers of GFP^+^ cells in the cortex at E15.5 (Figure 2G), double mutants had many more *Sst*^+^ cells than *Sp9* single mutants (Figure 3D). Moreover, more ectopic *Sst*^+^ cells accumulated in the ventral telencephalon in double mutants compared with single mutants (Figures 3B,C). *Erbb4* is mainly expressed in migratory and mature PV^+^ cortical interneurons (Fazzari et al., 2010; Mayer et al., 2018). We found that the percentage of *Erbb4*^+^ cells was reduced in the mutant cortical MZ, whereas it was greatly increased in the cortical SVZ (Figures 3E–I). Again, *Nkx2-1-Cre; Sp8/9-DCKO* mice had relative more *Erbb4*^+^ cells in the cortical SVZ and in the ventral telencephalon than *Nkx2-1-Cre; Sp9-CKO* mice (Figures 3E–I). This finding suggests that loss of *Sp8/9* function results in relatively more immature PV^+^ cortical interneurons that migrate in the cortical SVZ and that ectopically accumulate in the ventral telencephalon. *Npas1* is mainly expressed in cortical interneurons in the MZ, although a small number of interneurons in the cortical SVZ also express *Npas1* (Cobos et al., 2006; Stanco et al., 2014). We found that the percentage of *Npas1*^+^ cells in the cortical MZ were reduced, whereas it was greatly increased in the cortical SVZ of mutants compared with controls (Figures 3J–M).

**Figure 4:**
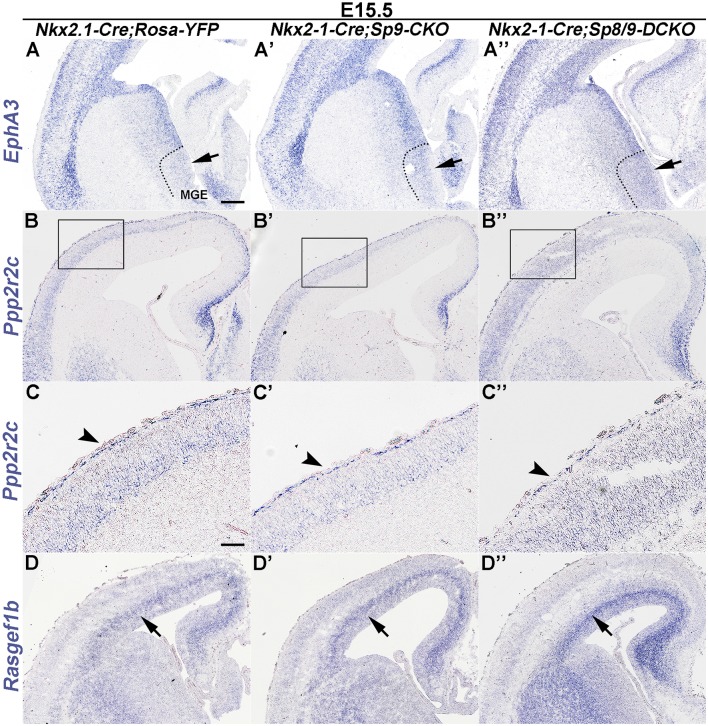
*In situ* RNA hybridization of *Epha3*, *Ppp2r2c* and *Rasgef1b* in coronal hemisections at E15.5. **(A–A″)** *EphA3* was upregulated in the mutant MGE (arrows). **(B–C″)** *Ppp2r2c* expression was reduced in the mutant cortical MZ (arrowheads). **(D–D″)** *Rasgef1b* expression was increased in the mutant cortical SVZ (arrows). Note that dysregulation of these genes was more prominent in *Sp8/9* double mutants than in *Sp9* single mutants. Scale bars: 200 μm in **(A)** for **(A–B″,D–D″)**; 100 μm in **(C)** for **(C–C″)**.

*Epha3* expression was increased in the mutant MGE at E15.5 (Figures 4A–A″), which explained why the LGE had very few MGE-derived cells, as enhanced Eph/ephrin signaling in the LGE VZ/SVZ increases the repulsive effect on migrating interneurons (Zimmer et al., 2008; Rudolph et al., 2010; Villar-Cerviño et al., 2015; Liu et al., 2018). Intracellular signaling molecule *Ppp2r2c* is mainly expressed in cortical interneurons in the MZ, whereas *Rasgef1b* is mainly expressed in cortical interneurons in the SVZ (Colasante et al., 2009; Antypa et al., 2011; Friocourt and Parnavelas, 2011). We found that the expression of *Ppp2r2c* in the cortical MZ was reduced, whereas the expression of *Rasgef1b* was greatly increased in the cortical SVZ of mutants compared with controls (Figures 4B–D″; Antypa et al., 2011). Notably, these phenotypes were more prominent in *Sp8/9* double mutants than in *Sp9* single mutants (Figures 4A–D″), further suggesting that *Sp8* supplements the role of *Sp9* in regulating MGE-derived cortical interneuron migration.

### PV^+^ Cortical Interneurons Are Severely Reduced in *Sp8/Sp9* Double Mutants

**Figure 5:**
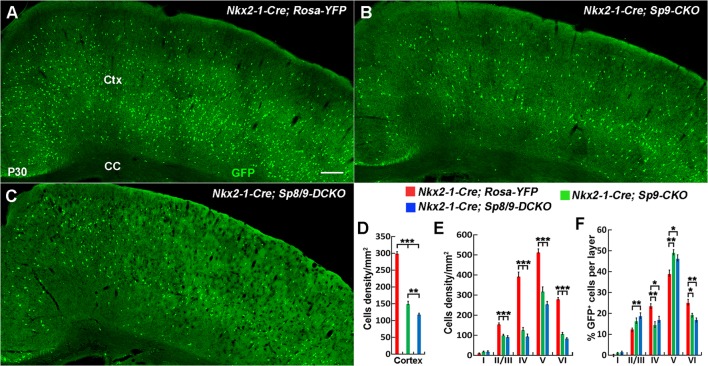
The number of MGE-derived cortical interneurons was significantly reduced in double mutants compared with single mutants and controls at P30. **(A–C)** GFP^+^ cells in the cortex of *Nkx2-1-Cre; Rosa-YFP* (control), *Nkx2-1-Cre; Sp9^F/F^; Rosa-YFP* (single mutant) and *Nkx2-1-Cre; Sp8^F/F^; Sp9^F/F^; Rosa-YFP* (double mutant) mice. **(D–F)** Quantified data showed that the density of GFP^+^ cells in the somatosensory cortex was reduced in single mutants and further reduced in double mutants compared with controls. Ctx, cortex; CC, corpus callosum. *P < 0.05; **P < 0.01; ***P < 0.001. Scale bars: 200 μm in **(A)** for **(A–C)**.

Analysis of the P30 neocortex showed profound MGE-derived cortical interneuron defects, especially PV^+^ cortical interneurons. We found that the density of MGE-derived cortical interneurons (GFP^+^ cells) in the somatosensory cortex was significantly reduced in *Nkx2-1-Cre; Sp8/9-DCKO* mice compared within *Nkx2-1-Cre; Sp9-CKO* mice and controls (Figures 5A–D). The density of GFP^+^ cells was also greatly reduced in each cortical layer (Figure 5E), but the percentage of GFP^+^ cells in cortical layers II/III and V was relatively increased in single and double mutants (Figure 5F).

**Figure 6:**
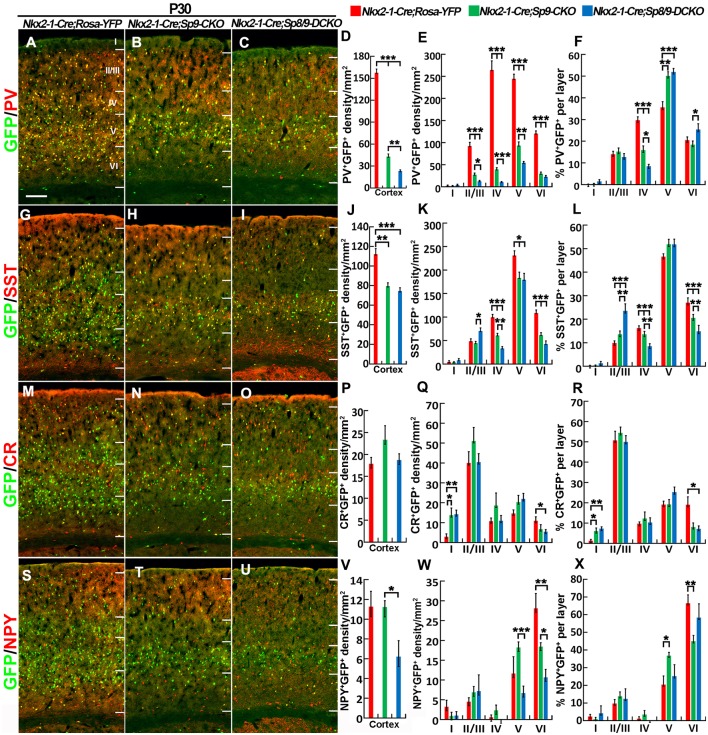
PV^+^ cortical interneurons are severely reduced in the *Sp8/9* double mutant cortex at P30. **(A–C,G–I,M–O,S–U)** GFP and cortical interneuron markers [PV, SST, calretinin (CR) and NPY] in double-immunostained coronal sections of the somatosensory cortex. **(D–F,J–L,P–R,V–X)** Quantified data showed that the density of PV^+^/GFP^+^ cells was severely reduced in double mutants compared with single mutants and controls. The density of SST^+^/GFP^+^ and NPY^+^/GFP^+^ cells was also reduced in the mutant somatosensory cortex, whereas CR^+^/GFP^+^ cells were less affected. *P < 0.05; **P < 0.01; ***P < 0.001. Scale bars: 200 μm in **(A)** for **(A–C,G–I,M–O,S–U)**.

We next quantified the density of GFP^+^/PV^+^, GFP^+^/SST^+^, GFP^+^/NPY^+^, and GFP^+^/CR^+^ interneurons in the somatosensory cortex (Figures 6A–X). The most prominent phenotype was severe loss of GFP^+^/PV^+^ cortical interneurons: *Sp8/9* double mutants showed ~80% reduction, and *Sp9* single mutants showed ~65% reduction (Figures 6A–E). GFP^+^/NPY^+^ cortical interneurons were also reduced in double mutants compared with single mutants or controls (Figures 6S–X), whereas GFP^+^/SST^+^ cells and GFP^+^/CR^+^ cells were less affected in double mutants (Figures 6G–K,M–R). In *Sp9* single and *Sp8/9* double mutants, the percentage of GFP^+^/PV^+^ cells was higher in cortical layer V, whereas the percentage of GFP^+^/PV^+^ cells and GFP^+^/SST^+^ cells was lower in cortical layer IV (Figures 6F,L), further indicating that *Sp8/9* not only regulates migration but also affects the layer distribution of MGE-derived cortical interneurons.

**Figure 7:**
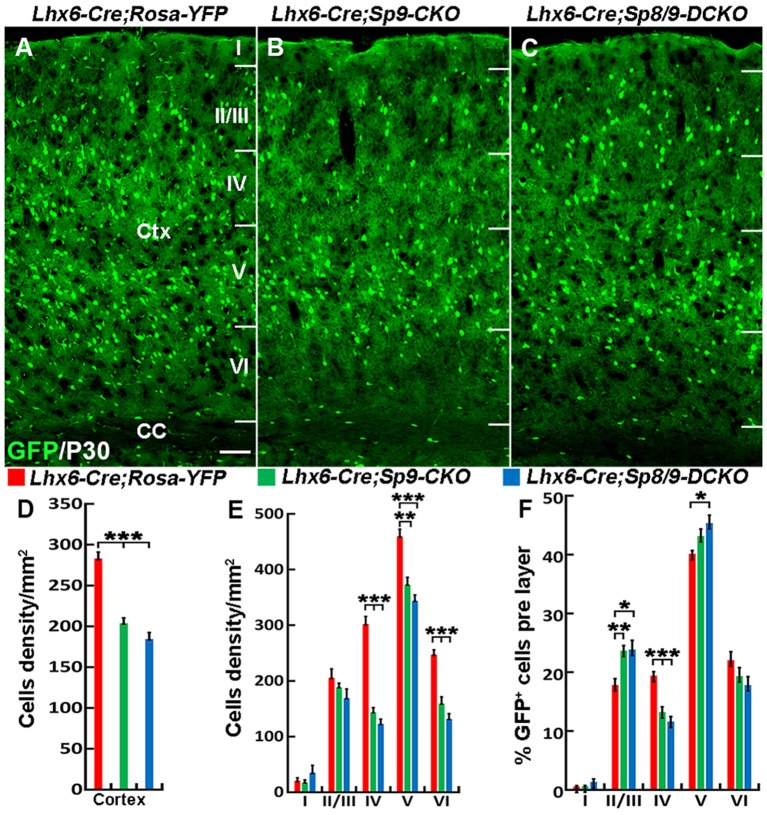
The number of MGE-derived cortical interneurons is greatly reduced in the somatosensory cortex of *Lhx6-Cre; Sp8^F/F^; Sp9^F/F^; Rosa-YFP* mice compared with *Lhx6-Cre; Rosa-YFP* and *Lhx6-Cre; Sp9^F/F^; Rosa-YFP* mice at P30. **(A–C)** GFP^+^ cells in the somatosensory cortex. **(D–F)** Quantified data from the above experiments. The density of GFP^+^ cells was significantly reduced in mutants compared with controls. Ctx, cortex; CC, corpus callosum. *P < 0.05; **P < 0.01; ***P < 0.001. Scale bars: 200 μm in **(A)** for **(A–C)**.

**Figure 8:**
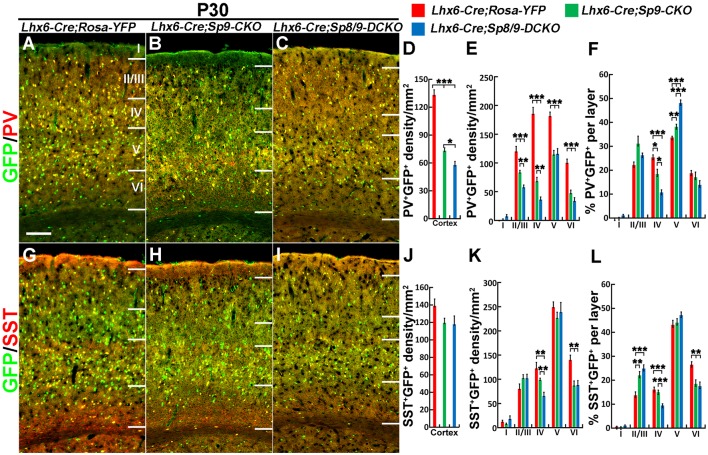
PV^+^ cortical interneurons are severely reduced in the *Lhx6-Cre; Sp8^F/F^; Sp9^F/F^; Rosa-YFP* somatosensory cortex. **(A–C,G–I)** GFP/PV and GFP/SST double-immunostained coronal sections. **(D–F,J–L)** The density of PV^+^/GFP^+^ cells was severely reduced in double mutants compared with single mutants and controls, whereas the density of SST^+^/GFP^+^ cells was less affected. *P < 0.05; **P < 0.01; ***P < 0.001. Scale bars: 200 μm in **(A)** for **(A–C,G–I)**.

Nkx2-1-Cre recombination occurs from the MGE VZ, the SVZ and the mantle zone (Xu et al., 2008). We, therefore, used the *Lhx6-Cre* transgenic line to conditionally knock out *Sp8/9* in postmitotic MGE-derived neurons (Fogarty et al., 2007; Nóbrega-Pereira et al., 2008) and quantified the density of GFP^+^, GFP^+^/PV^+^ and GFP^+^/SST^+^ interneurons in the somatosensory cortex. In general, loss of *Sp8/9* function in postmitotic MGE cells resulted in greatly reduced GFP^+^ and GFP^+^/PV^+^ cortical interneuron density (Figures 7A–F, 8A–L). Furthermore, an altered allocation of MGE-derived interneurons in the cortical layers was also observed in the mutants (Figures 7A–F, 8A–L), consistent with results of the *Nkx2-1-Cre* line knockouts.

## Discussion

The function of *Sp9* in the MGE has been described (Liu et al., 2018), but the function of *Sp8* in the MGE, which is highly homologous with *Sp9* and weakly expressed in the MGE mantle zone, has not yet been studied. Here, we found that the expression of SP8 in the MGE was significantly increased in *Sp9* null mutants. When we conditionally deleted both *Sp8* and *Sp9* in the MGE, the tangential migration of MGE-derived cortical interneurons was greatly inhibited, and the density of MGE-derived cortical interneurons, especially PV^+^ interneurons, was greatly reduced. These results suggest that *Sp8* plays a supplementary role to *Sp9* in regulating MGE-derived cortical interneuron development.

Previous studies have mainly focused on the function of *Sp8* in the LGE, which show that *Sp8* is important for the development of olfactory bulb interneurons and striatal medium spiny neurons (Waclaw et al., 2006; Liu et al., 2009; Li et al., 2011, 2018; Xu et al., 2018). SP8 is very weakly expressed in the MGE mantle zone (Ma et al., 2012; Vogt et al., 2014), but its function remains to be investigated. In the present study, we found that SP8 expression was upregulated in the *Sp9* mutant MGE, indicating that SP8 could compensate for SP9 function. Indeed, when we used the *Nkx2-1-Cre* transgenic line to conditionally knock out *Sp8/9*, the tangential migration of MGE-derived cortical interneurons was blocked, resulting in ectopic accumulation of interneuron-like cells in the ventral telencephalon. We propose that the loss of 80% of PV^+^ cortical interneurons was mainly due to defects in interneuron migration, as conditional deletion of *Sp8/9* in MGE neural stem cells using *Nkx2-1-Cre* lines and conditionally delete *Sp8/9* in MGE-derived postmitotic cells using *Lhx6-Cre* lines show a similar reduction of cortex interneurons. Furthermore, we observed that the apoptotic cell death in the control, single and double mutant cortices were at the same levels (data not shown), suggesting that *Sp8/9* do not affect the survival of MGE-derived cortical interneurons.

We suggest that this migratory deficit results from multiple mechanisms. In the double mutant neocortex, *Erbb4*^+^ immature PV interneurons mainly migrated in the cortical SVZ, whereas only a few migrated in the cortical MZ. Furthermore, we observed that more *Erbb4*^+^ cells ectopically accumulated in the ventral telencephalon. This finding explains why PV^+^ cortical interneurons were severely reduced in the double mutant cortex compared with the single mutant cortex and indicate that *Sp8* indeed has an important function in promoting MGE-derived cortical interneuron migration. The *Ppp2r2c* gene, encoding a subunit of protein phosphatase 2A, has a unique expression pattern in the embryonic mouse neocortex; interneurons in the cortical MZ express *Ppp2r2c*, but interneurons in the cortical SVZ do not (Colasante et al., 2009; Antypa et al., 2011; Friocourt and Parnavelas, 2011). *Rasgef1b* expression appeared to be limited to the cortical SVZ interneuron stream (Colasante et al., 2009; Antypa et al., 2011; Friocourt and Parnavelas, 2011). The *Rasgef1b* gene encodes a guanine nucleotide exchange factor for Ras family proteins and is a downstream target of *Arx* (Friocourt and Parnavelas, 2011). We found that the expression of *Rasgef1b* was significantly upregulated after removal of *Sp8/9*, whereas the expression of *Ppp2r2c* in the cortical MZ was significantly decreased. The dysregulation of these two genes may explain the great increase in the proportion of MGE-derived GFP^+^ cells in the cortical SVZ. Notably, loss of *Sp8/9* in the dorsal LGE also induced a block in tangential migration from the LGE to the olfactory bulb (Li et al., 2018; Guo et al., 2019). Thus, we propose that *Sp8/9* genes might play a general role in regulating the tangential migration of telencephalic interneurons.

## Author Contributions

GT and ZL performed all experiments and analyses. YW, XS, SW and HD helped conduct some experiments and analyze the data. ZY, and YY helped guide the project and discussed the results. ZY, ZX, YY and GT wrote and edited the manuscript.

## Conflict of Interest Statement

The authors declare that the research was conducted in the absence of any commercial or financial relationships that could be construed as a potential conflict of interest.
